# Reduced contextual uncertainty facilitates learning what to attend to and what to ignore

**DOI:** 10.3758/s13414-024-02945-z

**Published:** 2024-09-10

**Authors:** Chris Jungerius, Sophie Perizonius, Heleen A. Slagter

**Affiliations:** 1 Swammerdam Institute for Life Sciences, University of Amsterdam, Amsterdam, The Netherlands; 2 Department of Experimental and Applied Psychology, Vrije Universiteit, Amsterdam, The Netherlands; 3 Institute for Brain and Behavior, Amsterdam, The Netherlands

**Keywords:** Visual search, Attentional capture, Contextual cueing, Statistical learning, Distractor suppression

## Abstract

Variability in the search environment has been shown to affect the capture of attention by salient distractors, as attentional capture is reduced when context variability is low. However, it remains unclear whether this reduction in capture is caused by contextual learning or other mechanisms, grounded in generic context-structure learning. We set out to test this by training participants (*n* = 200) over two sessions in a visual search task, conducted online, where they gained experience with a small subset of search displays, which significantly reduced capture of attention by colour singletons. In a third session, we then tested participants on a mix of familiar and novel search displays and examined whether this reduction in capture was specific to familiar displays, indicative of contextual cueing effects, or would generalise to novel displays. We found no capture by the singleton in either the familiar or novel condition. Instead, our findings suggested that reduced statistical volatility reduced capture by allowing the development of generic predictions about task-relevant locations and features of the display. These findings add to the current debate about the determinants of capture by salient distractors by showing that capture is also affected by generic task regularities and by the volatility of the learning environment.

## Introduction

In recent years, it has become increasingly clear that brains continuously try to predict what sensory signals are likely informative to the task at hand based on past experience and statistical structure in the environment (de Lange et al., 2018; Friston, 2009), and that such statistical learning affects how attention is oriented (Jiang, 2018; Theeuwes et al., 2022). For example, many studies have now shown that attention is driven towards locations where participants have learned targets are likely to be found and away from likely distractor locations (Geng & Behrmann, 2005; Goschy et al., 2014; Miller, 1988; van Moorselaar & Slagter, 2019; Wang & Theeuwes, 2018). These findings indicate that attention incorporates memories about the statistical structure of information, providing the brain with aa mechanism to predict which aspects of the environment are likely relevant for goal-directed action in the future and which can be safely ignored. One influential proposal, supported by recent empirical findings, is that statistical learning allows the brain to proactively suppress distracting information, preventing it from capturing attention (Luck et al., 2021). For example, using a search-probe task in which the colour of the singleton distractor was fixed and hence predictable across trials, Gaspelin et al. (2015) showed that initial attention to the singleton was below baseline, indicative of proactive suppression.

We recently demonstrated that whether or not individuals can learn to ignore distracting information and prevent attentional capture, is dependent on contextual uncertainty (Jungerius et al., 2022). Using a similar search-probe task (Gaspelin et al., 2015), but with eight search items, we found that only when the number of visual search configurations (possible combinations of shapes with a fixed spatial layout) shown to participants was reduced to a small subset of the possibility space (from ~1,160 to 24 configurations), participants demonstrated the ability to overcome attentional capture by a salient distractor with a predictable colour. This disappearance of capture notably only emerged in session two, after participants had already been exposed to the set of restricted configurations for one session, and was not observed in a condition of high statistical volatility (which included all possible search configurations). These findings demonstrate that contextual uncertainty can affect learning to ignore, and align with another, mostly separate literature showing that spatial contextual learning can strongly guide attention (Gao et al., 2023; Ogawa et al., 2007; Sisk et al., 2019; Vadillo et al., 2021) and more recent work in that domain demonstrating that the speed at which spatial contextual cueing effects emerge is dependent on the assumed stability of the environment in which learning takes place, such as the relative proportion of repeated versus non-repeated search displays (Zinchenko et al., 2018, 2024). They also fit with another literature that shows that (expected) volatility of a reward environment can affect the saliency assigned to information for predicting future outcomes and thereby the learning speed (e.g., Behrens et al., 2007).

In this preregistered study, we set out to directly test how contextual uncertainty affects the ability to ignore salient distractors. One possibility is that the acquired ability to reduce attentional capture in contexts with low contextual uncertainty reflects contextual learning, i.e., due to the repeated configurations. While it is well known that contextual learning about target locations strongly affects search (Chun & Jiang, 1998), few studies have so far investigated effects of contextual learning about distracting or task-irrelevant stimuli, and in all these studies, the spatial layout of the search elements was predictive of the distractor location(s). Their findings suggest that spatial context-dependent distractor learning can also facilitate performance. In one study, suppression of distractors at a highly probable location was shown to be contingent upon the specific spatial configuration of the search display (Gao et al., 2023). In another study (Vadillo et al., 2021), it was found that learning which locations in a search context do not contain targets facilitated search performance. In our past study in which we manipulated contextual uncertainty by restricting the number of shape combinations, while their spatial layout was kept the same, we found that low contextual uncertainty reduced attentional capture (Jungerius et al., 2022). It is hence conceivable that in a restricted set of search contexts, these feature contexts start to serve as attentional cues as participants become familiar with them.

An alternative possibility is that reduced environmental volatility experienced by the participant does not affect contextual learning, but enhances sensitivity to statistical structure in the sensory input, facilitating distractor learning (Slagter & Moorselaar, 2021). As pointed out above, it is well known that environmental volatility can affect learning speed (e.g., Behrens et al., 2007). When contextual volatility is very high, it is possible that the brain considers the sensory input not representative of a stable environment, reducing new learning, while when contextual volatility is low, it is considered more informative, enhancing the learning speed. Interestingly, recent work suggests that reduced capture by salient distractors observed using search-probe tasks with relatively low contextual variability may not simply reflect learning to ignore a distractor with a predictable colour, but global feature enhancement of all items with the possible (predictable) target colour (Oxner et al., 2023; van Moorselaar et al., 2023). Contextual uncertainty could affect both distractor- and target-based predictive learning.

To determine how contextual uncertainty affects learning to ignore a salient distractor, and dissociate between these two possibilities, we used an experimental procedure often used in the contextual cueing literature (Chun, 2000): after two sessions in which participants became acquainted with a small set of repeated display configurations (as in our previous study), we mixed familiar and novel search configurations in a third session to test whether the learned reduction in capture participants were expected to exhibit in session 2 was specific to the search configurations they had become familiar with, or if it would generalise to new search configurations.

We hypothesised that if contextual cueing was the cause of the reduction in capture, the reduced contextual variability, caused by repeated presentation of specific search displays, would reduce or even abolish initial attentional capture over time, replicating our initial study findings (Jungerius et al., 2022). Specifically, we predicted that attentional capture, as measured by the difference in probe letter recall between singleton- and non-singleton distractor locations, would reduce or disappear across the first two sessions. In addition, we expected that this reduction in, or elimination of, attentional capture would be specific to the familiar contexts in session 3. In other words, we predicted greater attentional capture, as reflected in higher probe letter recall at singleton-distractor compared to non-singleton distractor locations, only for novel search configurations. Note that in this case, the contextual cuing effect would not be location based (as in Gao et al., 2023), but based on the combination of search element shapes. If we instead observed a reduction in capture which generalised to the novel contexts, this would suggest that the reduction in capture was primarily driven by context-independent predictive learning, such as global enhancement of target features (Oxner et al., 2023).

## Methods

This experiment followed a preregistered design and analysis path (https://osf.io/d69fk)

### Participants

Based on estimates of required participant numbers (Brysbaert, 2019) and a simulation-based power analysis using our previous design to estimate effect sizes (DeBruine & Barr, 2021; Green & MacLeod, 2016), we collected data from 200 participants in total (86 women, 114 men, mean age of 26.7 years), who were recruited through the online platform Prolific. They were screened for age (18–40 years), normal or corrected-to-normal vision, and normal colour vision. All participants provided informed consent prior to participating in the experiment. The experiment was approved by The Scientific and Ethical Review Board (VCWE) of the Faculty of Behavior and Movement Sciences of the Vrije Universiteit.

### Stimuli, design and procedure

Our experimental setup was repeated from our previous work using this paradigm (Jungerius et al., 2022). The experiment was coded in JavaScript using the JsPsych library (de Leeuw, 2015) and conducted online trough Prolific.

At the start of the experiment, the size and resolution of the participant’s display and the distance participants were sitting from their display was determined using a ‘virtual chinrest’ (Li et al., 2020). These estimates were used to scale the stimuli used in the experiment, which ensured that stimuli would have the same size in units of visual angle for all participants.

On each trial, first a fixation dot was presented for 500 ms, followed by an eight-item search display. Search displays contained a circle, a diamond, three squares and three hexagons. The target shape (circle or diamond; counterbalanced across participants) and target colour (green or red; counterbalanced across participants) were held constant for the entire experimental session. In singleton-present trials, one of the distractor shapes had a unique colour (i.e., red or green, counterbalanced across participants). Stimuli were 2.4 × 2.4 degrees visual angle in size. They were arranged evenly spaced with the centre of each stimulus on a circle around fixation with a radius of 2.5 degrees visual angle.

**Fig. 1:**
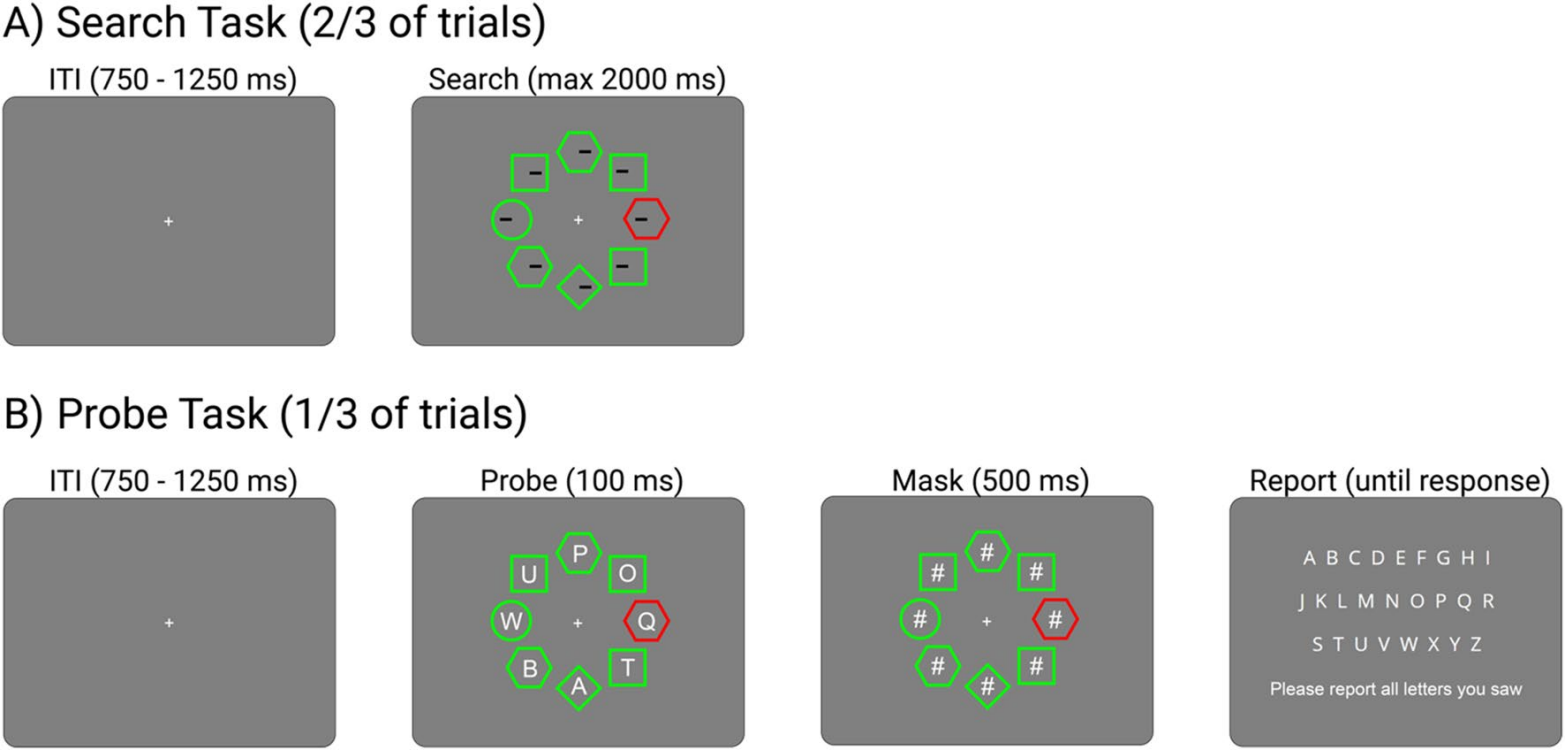
Task structure for the two types of tasks used during the experiment. **A:** In two-thirds of all trials, participants performed a search task, where they had to report the orientation of a line segment within a target (here a diamond). **B:** In one-third of all trials, letters were shown simultaneously to search display onset, followed by a mask. Participants were asked to report all letters they detected. Singleton distractors and targets always appeared on the four cardinal locations (top, bottom, left and right)

During the experiment, participants performed two types of tasks, which were randomly interspersed within blocks: search trials and probe trials (Fig. 1). In *search trials* (two-thirds of trials), each shape contained a line segment to the left or right of the shape’s centre, and participants were tasked with indicating whether a line inside the target shape was more towards the left or right side of that shape, within a time limit of 2 s. The search display disappeared upon response or after 2 s. In *probe trials* (one-third of trials), the search display only appeared for 100 ms, with one letter displayed inside each shape of the search display. This was followed by a mask consisting of a hash sign at each letter location for 500 ms, to prevent influence by iconic memory. Participants were then prompted to report all letters they remembered seeing using the keyboard. Letters were chosen randomly without replacement from all 26 letters in the Latin alphabet.

During an experimental run, participants first received instructions on the visual search task before practising with it until they responded accurately in 70% of trials in a practice block of 32 trials. Subsequently the probe task was introduced, and participants again practised with blocks of 33 trials (11 probe trials and 22 search trials) to familiarise themselves with the probe task and the proportion of probe-to-search task trials, until they reached 70% accuracy in the search task (note that accuracy in the probe task was not tested, as the perception of probe is expected to be difficult and the task does not have a simple ‘correct’ response like the search task does). The main experiment would then begin.

To reduce the contextual uncertainty of the task, we manipulated the variability of the search display, following Jungerius et al. (2022): targets and distractors only appeared on the four cardinal locations. For each of the four possible target locations, three search display configurations were randomly drawn from the range of possible configurations that could be formed from the shapes used in the display. In other words, three unique arrangements of shapes (consisting of squares, hexagons, circle and diamond) were selected for each of the four possible target locations. A given participant would only ever be shown displays drawn from this restricted set. This meant that the range of configurations a participant would be exposed to in sessions 1 and 2 would total 12. In addition, for a given context or arrangement of shapes, the singleton distractor would always appear in the same location. Therefore, each of the 12 configurations participants would see was predictive of a unique combination of target and singleton locations. Each of these configurations was shown equally often to participants, and each configuration had the same probability of being used in a singleton-present or singleton-absent trial, meaning that the configurations were not themselves predictive of singleton presence. The line segments or letters used for search and probe trials were not fixed for a given configuration.

Participants performed the task with this restricted set of configurations for two sessions on two separate days. In a third session, half the trials were drawn from the restricted set, with the other half consisting of ‘novel’ configurations new to the participant. In these novel configurations, targets and distractors could also only appear at the four cardinal locations.

The first two sessions consisted of four blocks of 96 trials (total: 384 trials), and the length of the last session was six blocks of 96 trials (total: 576 trials), to allow for sufficient data collection for a direct comparison of novel and familiar trials. Two-thirds of the trials were search trials (64 per block), and one-third of the trials were probe trials (32 per block). A uniquely coloured singleton distractor was present in half of the search trials and all probe trials.

To measure the degree to which participants had learned to recognise the set of contexts they were exposed to in the first two sessions, at the end of the third session, participants were presented with eight novel and eight familiar search display configurations and asked to rate them on a scale from ‘completely unfamiliar’ to ‘completely familiar’ using an on-screen response slider.

### Analysis

All analyses were preregistered unless specified otherwise, and performed in R (R Development Core Team, 2018). Mixed models and ANOVAs were fitted using the *lme4* and *afex* packages (Bates et al., 2015; Singmann et al., 2023), and Bayesian models were fitted using the *brms* package (Bürkner, 2018).

#### Probe task

As in previous work (Gaspelin et al., 2015; Jungerius et al., 2022), our main outcome of interest was the detection of letters during the letter probe task, as this allowed us to selectively study attentional orienting during the early stage (i.e., the first 100 ms) of visual search. In all analyses, attentional capture was defined as the difference in probe letter recall at singleton versus non-singleton (control) locations. Probe letter recall at non-singleton locations was calculated by averaging probe letter recall across the two cardinal locations that did not contain a target or singleton.

Participants with incomplete or corrupted data or 0% letter recall were excluded from analysis (eight participants).

We first established the initial presence of attentional capture in the first half of session 1 (before extensive contextual learning), by contrasting probe letter recall at singleton versus non-singleton locations with a generalised linear mixed-model analysis (GLMM) with a binomial (logit) link function.

Our first main prediction that attentional capture would reduce between session 1 and 2 was tested with a GLMM with a binomial (logit) link function, testing the recall ~ session * location + (session * location | subject) model (confined to the first two sessions of the experiment). Of interest here is the session * location interaction, tested for significance using a likelihood ratio test against a model without an interaction term. Model contrasts were used to identify significant differences between locations (control, distractor) within each session.

Next, to test whether the loss of capture would be specific to familiar contexts, or would generalise to novel contexts, a GLMM with a binomial (logit) link function was used, testing the recall ~ novelty * location + (novelty * location | subject) model. Of interest here is the novelty * location interaction, tested for significance using a likelihood ratio test against a model without an interaction term. Model contrasts were used to identify significant differences between locations (control, distractor) within each condition (familiar, novel). In case of a significant interaction, planned comparisons (model contrasts) were conducted to establish the presence or absence of capture separately in trials with a familiar and in trials with a novel search configuration.

Bayesian hierarchical model analogues of the models described above were also fitted to the data, and the posterior marginal likelihood of the data for each model was compared to obtain measures of relative evidence for and against the presence of an interaction term in terms of Bayes factors (Gronau et al., 2020; Kass & Raftery, 1995).

In line with Barr et al. (2013), we adjusted the model structure in the above analyses from our preregistration to include a random slope for all effects per participant. In the event of a singular model fit, random slopes with the smallest variance component were dropped until models fit without such issues. In practice, this only led to the dropping of the random slope for novelty in the GLMM for session 3 (final model structure: recall ~ novelty * location + (location | subject))

In an exploratory analysis, to test the extent to which participants learned to selectively attend to potential target locations, we also examined the difference in detection of letters at non-singleton distractor locations on the cardinals (where a target could appear) and diagonals (where a target would never appear) using a mixed model with a structure comparable to the models listed above.

### Search task

We also examined attentional capture at the level of reaction times in search trials. As preregistered, participants with an error rate that differed by 2.5 standard deviations from the mean error rate were excluded from analysis (sessions 1 and 2: nine participants; session 3: six participants). The remaining incorrect and timed-out trials were excluded from analysis (sessions 1 and 2: incorrect trials: 2.9% of trials, timed out trials: 0.1% of trials; session 3: incorrect trials: 2.5% of trials, timed out trials: < 0.001% of trials). Trials with a reaction time of less than 200 ms were also excluded from analysis (< 0.001% of trials in both conditions).

The reaction time data were then filtered through a two-step outlier removal process; trials were trimmed on the basis of a cut-off value 2.5 standard deviations from the mean per participant. Next, participants whose average reaction time differed by 2.5 standard deviations from the conditional mean were excluded from analysis (sessions 1 and 2: within-participant trimming: 8.35% of trials, between-participant trimming: five participants; session 3: within-participant trimming: 5.29% of trials, between-participant trimming: seven participants).

The difference in the effect of singletons on reaction time between sessions 1 and 2 were tested with a repeated-measures ANOVA on the dependent variable reaction time, with within-subject factors distractor presence with two levels (true and false) and session with two levels (1, 2).

The difference in the effect of singletons on reaction time between novel and familiar contexts was tested with a repeated-measures ANOVA on the dependent variable reaction time in session 3, with within-subject factors distractor presence with two levels (true and false) and context familiarity (familiar and novel).

### Contextual learning

To detect whether the familiarity ratings participants provided at the end of the experiment differed significantly between novel and familiar search displays, their familiarity judgements were tested with a paired t-test comparing the averaged familiarity ratings for novel and repeated configurations across subjects.

## Results

### Probe task

Looking at results from the first half of session 1 confirmed that capture was present during the first half of session 1, when participants were not yet as familiar with the search contexts: a GLMM with location as a predictor fit the data significantly better than an intercept-only model (likelihood ratio test: Chi^2^(1) = 32.62, *p* < .001), with an odds ratio for detection at the singleton over control locations of 1.29 (*z* = 6.032, *p* < .001). This result confirms that singleton distractors initially captured more attention than non-singletons, as reflected by enhanced letter recall.

**Fig. 2:**
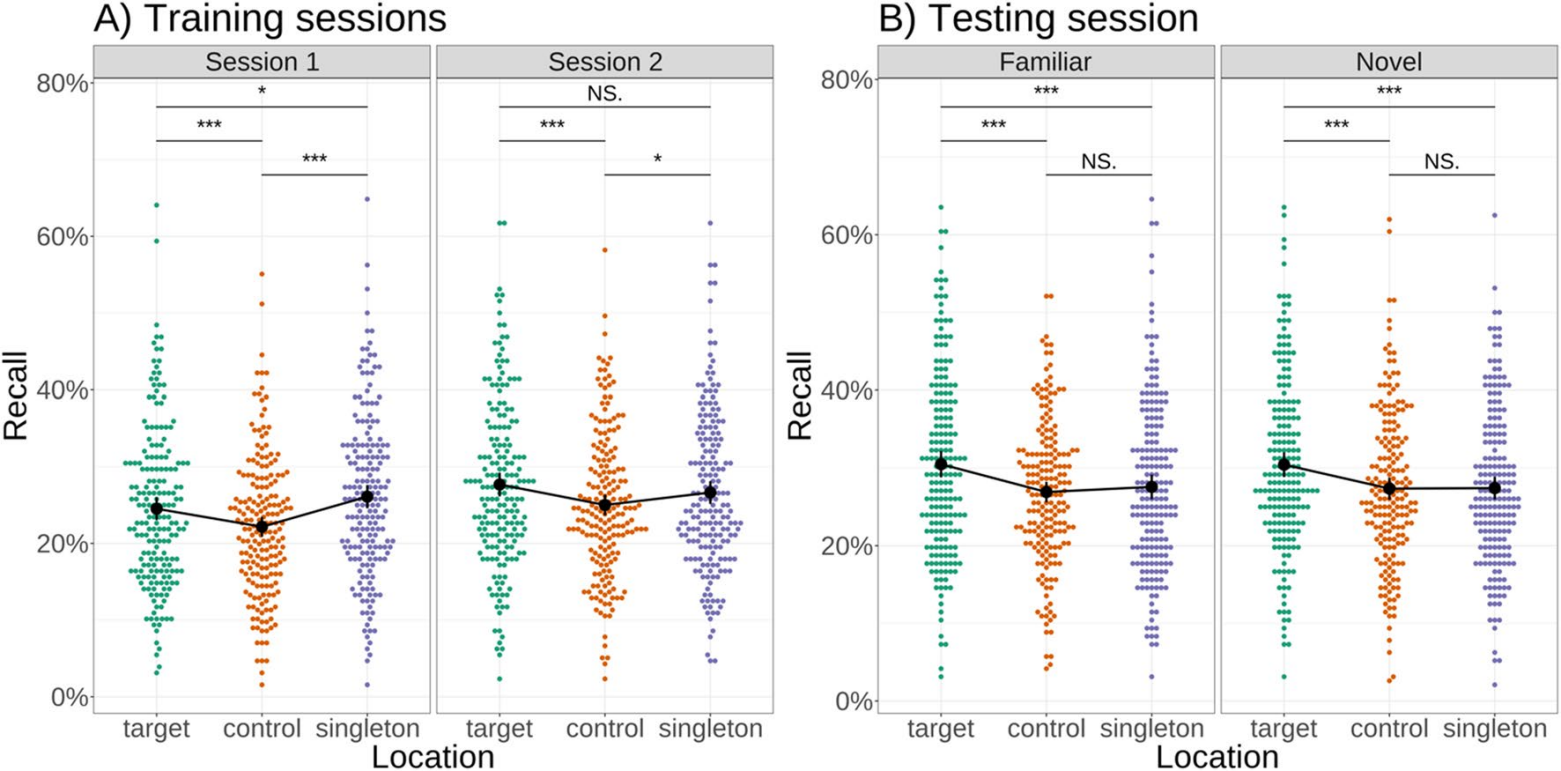
Early attentional capture by singleton distractors reduced significantly over two sessions in a reduced contextual variability condition. **A:** Shown is a comparison of recall of probe letters at target, control and singleton distractor locations by participants during the first two sessions. The difference in recall between letters shown at control and singleton locations (i.e., attentional capture) went down significantly between sessions. **B:** This learned reduction in capture generalises to novel configurations: comparing recall of probe letters at control and singleton distractor locations by participants for novel and familiar displays during the third session, there is no capture (no significant difference in recall between the two locations) in either familiar or novel displays. *NS* not significant, * *p* < .05, *** *p* < .001

Capture was present in session 1 (odds ratio for detection at the singleton over controls: 1.25, *z* = 6.366, *p* < .001), and present, but strongly reduced, in session 2 (odds ratio for detection at the singleton over controls: 1.09, *z* = 2.327, *p* = .020) (Fig. 2A). This reduction in capture by singleton distractors over time was significant, as shown by a significant interaction between session and location (likelihood ratio test between model with and without interaction term: Chi^2^(1) = 16.20, *p* < .001). Comparing Bayesian analogues of these models, we found a Bayes factor of 556 in favour of the model with an interaction term between session and location, which can be interpreted as decisive evidence in favour of the inclusion of an interaction term (Kass & Raftery, 1995). As expected, we thus replicated our previous finding that, in search tasks with low contextual uncertainty, capture by singleton distractors is significantly reduced over time. We have shown previously that this reduction of capture does not occur when contextual uncertainty remains high (Jungerius et al., 2022).

Contrary to our main prediction that the observed reduction in capture can be explained by contextual learning, comparing the results of novel versus familiar configurations in session 3, however, revealed no difference in capture between the two conditions (Fig. 2B) – the interaction between novelty and location was not significant (likelihood ratio test between full and restricted model: Chi^2^(1) = 1.18, *p* = 0.276), with no significant capture in either condition (odds ratio for detection at the singleton over control locations in familiar trials: 1.033, *z* = 1.025, *p* = .306, novel trials: 1.000, *z* = 0.010, *p* = .992). Comparing Bayesian analogues of these models, we found a Bayes factor of 4.12 in favour of the model without an interaction term, which can be interpreted as substantial evidence in favour of the exclusion of the interaction term. These findings indicate that the observed reduction in capture was context-independent.

### Exploratory analysis of letter recall at cardinal and diagonal locations

During the analysis of the letter recall data, we observed that, in line with our previous study (Jungerius et al., 2022), the reduction in capture between sessions 1 and 2 was to a large extent driven by an increase in recall of letters at cardinal non-singleton distractor locations, while detection of letters at the location of the singleton distractor remained at approximately the same level between sessions. We hypothesised that this increase might be due to the development of generic predictions about the task relevance of different elements of the search display, not only in the colour dimension, but also in terms of their location. To investigate the source of this increase in recall, we performed an exploratory analysis where we compared letter recall at task-relevant cardinal and task-irrelevant diagonal non-singleton locations.

**Fig. 3:**
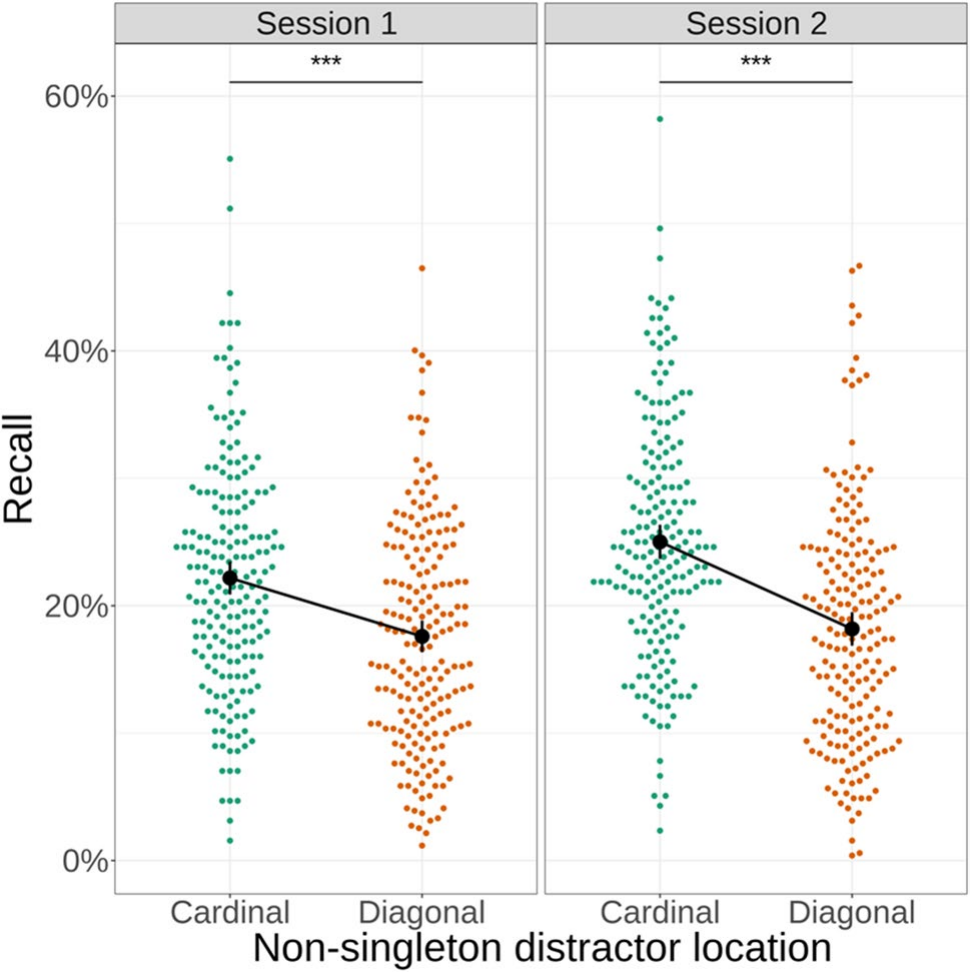
Diagonal non-singletons are recalled less often than cardinal non-singletons: Shown is a comparison of participant’s recall performance for letter probes presented at diagonal and cardinal non-singleton distractor locations in the first two sessions of the experiment. Letter probes at cardinal locations were detected significantly more often in both sessions, with a significant increase between sessions. *** *p* < .001

We found that over time, letter recall increased selectively for those locations that could potentially contain the target shape and that contained a shape of the target colour (Fig. 3) (a significant main effect of non-singleton location (cardinal, diagonal): Chi^2^(1) = 130.53, *p* < .001) and significant interaction between non-singleton location (cardinal, diagonal) and session (Chi^2^(1) = 24.24, *p* < .001), reflecting higher recall of letters at cardinal locations in session 2 compared with session 1 (odds ratio for recall of letters at cardinal vs. diagonal locations in session 1: 1.41, *z* = 10.98, *p* < .001; session 2: 1.62, *z* = 13.844, *p* < .001). This suggests that the increase in recall of cardinal non-singleton locations reflects a re-orienting of attention towards elements that are known to be task-relevant in terms of both colour and location.

### Search task

**Fig. 4:**
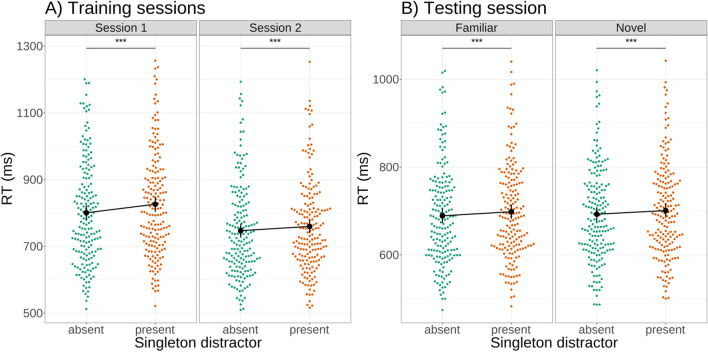
Attentional capture as indexed by reaction time reduced significantly over time: shown is a comparison of participant reaction times in singleton-present and singleton-absent trials for the first two sessions of the experiment (**A**). While capture remained in both sessions, it reduced significantly over time. **B:** There was no difference in attentional capture for novel and familiar displays. Comparing participant reaction times to singleton-present and singleton-absent trials for familiar and novel displays in the third session of the experiment, capture was similarly present in both conditions. *** *p* < .001

At the search reaction time level, singleton presence consistently slowed performance in the first and second session (Fig. 4A), as shown by a repeated-measures ANOVA (main effect of distractor presence: *F*(1,180) = 93.859, *p* < .001). This effect was significantly reduced in session 2 compared to session 1 (significant interaction between distractor presence and session: *F*(1,180) = 19.764, *p* < .001, difference in marginal means for session 1: 25.7, *t*(180) = 9.723, *p* < .001, difference in marginal means for session 2: 12.6, *t*(180) = 5.563, *p* < .001), suggestive of a reduction in attentional capture by singletons in session 2.

Comparison of singleton effects in the novel and familiar conditions in session 3 showed a significant main effect of novelty (*F*(1,181) = 6.759, *p* = .010), reflecting generally faster responses in familiar contexts. However, participants showed no significant difference in attentional capture between the two conditions (Fig. 4B) (no significant interaction between familiarity and distractor presence, *F*(1,181) = 0.193, *p* = .661), with capture overall remaining at a level slightly below that observed in session 2 (significant main effect of capture: *F*(1,181) = 36.49, *p* < .001, marginal mean difference between overall distractor present and absent conditions: 8.75, *t*(181) = 6.041, *p* < .001).

This pattern of results is largely similar to that observed in the probe trials. Although the modulation of capture over two sessions as measured by search RT appears smaller than the effect of early capture as indexed by probe letter recall, this finding is in line with previous work using this paradigm (Stilwell & Gaspelin, 2021; Wang & Theeuwes, 2020), which has shown a dissociation between probe recall and reaction time as indices of capture. This dissociation may reflect the fact that search RT reflects the summation of many different processes, not just initial capture (as letter probe recall does), such as filtering costs imposed by the singleton (Becker, 2007; Folk & Remington, 1998)*.*

### Contextual learning

Comparing familiarity ratings between novel and familiar contexts, we found a significant difference between novel and familiar contexts, with participants rating familiar contexts as slightly more familiar than novel contexts (*z* = 2.92, *p* = .0036).

## Discussion

We previously found that the ability to prevent attentional capture by highly physically salient distractors is strongly influenced by contextual uncertainty (Jungerius et al., 2022), a factor that has so far not been considered by any main theory of attentional capture as an important determinant of capture (Luck et al., 2021). Here, we replicate and extend this finding by examining whether the reduction in capture observed in a condition of low contextual uncertainty is due to contextual learning. We show that the elimination of attentional capture was not specific to familiar contexts, as salient distractors also no longer captured attention in novel contexts during session 3. Therefore, the observed reduction in capture cannot be attributed to contextual learning.

Our inability to find a significant effect of contextual learning is unexpected given that contextual learning typically develops automatically and rapidly (Gao et al., 2023; Sisk et al., 2019). In typical contextual learning tasks, contextual learning is based on the spatial configuration of the search elements (Sisk et al., 2019), which was not possible in our task, as all search configurations had the same circular spatial layout. Moreover, shape was not a defining target feature, while our task permitted learning about the target colour and its possible locations (the four cardinal locations). This may have reduced shape-based contextual learning. There is also some evidence that the presence of a singleton distractor can reduce contextual learning (Conci & von Mühlenen, 2009). It is thus possible that our specific search task did not create the optimal conditions for contextual learning.

If not contextual cueing, what, then, might account for the observed changes in early attentional orienting? We propose that the reduced contextual uncertainty may have facilitated the development of generic predictions about what elements of the search display were generally (i.e., across search configurations) task-relevant and which were not. In our task, the target could ever only appear at the cardinal locations, and furthermore, as in past work using this task (e.g. Gaspelin et al., 2015; Oxner et al., 2023; Wang & Theeuwes, 2020) the target and non-singleton distractor colour were the same and fixed for a given participant (e.g., green), while the singleton distractor had a unique colour that was also fixed across trials (i.e., red). It is thus possible that participants learned that anything, for example, green at the four cardinal locations was potentially relevant, and anything red was not. This possibility is supported by the exploratory analysis of letter recall at task-relevant cardinal and -irrelevant diagonal locations, which showed that letter recall for letters at cardinally placed non-singletons selectively increased as compared to letters at diagonal non-singletons, in line with past work showing how participants are able to focus their attention on locations that are more relevant to the current task (Geng & Behrmann, 2005). Yet, such an increase in letter recall was not observed for the cardinal, task-relevant location that contained a shape in the singleton colour. Given that capture is expressed as the difference in letter recall between singleton and non-singleton cardinal locations, the observed reduction in capture was to a large part driven by enhanced letter recall at the non-singleton locations that contained shapes in the target colour (i.e., in baseline letter recall). This raises the possibility that global target colour enhancement, albeit specific to the cardinal, possible target locations, contributed to the observed effects. This could also explain why capture disappeared in novel contexts in session 3: here, too, attention may have been selectively oriented towards the cardinal locations with a shape of the target colour. Rather than contextual learning, a context with low contextual uncertainty may have in other words allowed participants to pick up on generic predictive structure in the task, which may have induced an attentional strategy of attending to anything, for example, green at the four cardinal locations.

As noted in the *Introduction*, findings from a recent study provide direct support for the possibility that the observed reduction in capture over time reflects global colour enhancement. Using a similar search-probe task with search displays of set size 4 (i.e., a condition with relatively low contextual uncertainty), Oxner et al. (2023) found that the amount of capture observed varied as a function of target-non-singleton colour similarity: the more similar the non-singletons were in colour to the target, the more letter recall at non-singleton locations increased, and conversely, capture (the difference in letter recall between singleton and non-singleton locations) decreased. Oxner et al. argued that therefore the previously reported reduction in attentional capture reported in studies using a similar search-probe task with predictable target and singleton colours does not reflect proactive suppression, as is commonly assumed (Gaspelin & Luck, 2018; Luck et al., 2021), but rather global target colour enhancement. Our findings are in line with this proposal and moreover suggest that feature-based contextual variability may affect the development of global feature attention, similarly to how it has been shown to affect the speed at which effects of spatial contextual cueing on spatial attention arise (Zinchenko et al., 2018, 2024).

Our data leave unresolved whether proactive suppression may have also contributed to the observed reduction in capture, next to global target colour enhancement. Participants likely also learned that anything, for example, red (the singleton) at the cardinal locations was not task-relevant (see also Vadillo et al., 2021), as otherwise one may also expect enhanced letter recall at the singleton location due to absolute target location learning. The fact that letter recall at singleton locations did not increase over time could indicate global target colour enhancement, distractor colour suppression, or both. Notably, a recent ERP study found that the Pd, an ERP component typically associated with distractor suppression (Gaspelin et al., 2023), was reduced in amplitude when the target colour was not predictable compared to when it was predictable, despite the fact that in both conditions, the distractor colour was fixed and hence always predictable (van Moorselaar et al., 2023). This suggests that target expectations can also account for reductions in attentional capture observed in conditions in which the target and distractor colour are both predictable, as in our study. Of further relevance, another recent study found that the Pd reflects selection of non-target locations (opposite to the singleton distractor with a predictable colour), not distractor suppression (Kerzel & Huynh Cong, 2023). Future studies using EEG are necessary to better understand to what extent brains can proactively suppress distracting information based on past experience, or mainly engage in target feature upweighting.

As mentioned above, in our previous study (Jungerius et al., 2022), when contextual variability was high (all search configurations were possible), we found a general increase in letter recall across two sessions at all task-relevant cardinal locations, regardless of shape or colour, indicative of absolute target location learning. The fact that we did not find enhanced letter recall at the singleton location over time in the current study, but only at the non-singleton and target locations, argues against an interpretation of our findings simply in terms of absolute target location learning. Instead, they suggest that under low statistical volatility, more complex predictions can be formulated that combine target location foreknowledge with target colour foreknowledge to bias attention towards possible targets (Saenz et al., 2002) and away from distractors – in this case, because of a net shift of attention towards the target colour (e.g., green). Albeit an indirect comparison, given that we previously did not observe this effect in a context of high contextual variability, this suggests that such integration of colour and location foreknowledge is dependent on (the estimated) statistical volatility of the search environment. Indeed, a largely separate literature shows that learning is dependent on (assumptions about) the uncertainty of the task context (Mathys et al., 2011; Zinchenko et al., 2018, 2024). It is notable in this respect that previous studies showing alleged proactive suppression typically used search tasks in which the target and non-singleton colours were also the same and fixed, and in which contextual uncertainty was relatively low, as the search display only contained four or six items (e.g., Gaspelin et al., 2015). In general, our findings illustrate the importance of taking contextual uncertainty, as well as of global feature enhancement, into account in the study of distractor suppression (Wöstmann et al., 2022). They also highlight the fact that brains continuously try to predict what aspects of the environment are informative for goal-directed behavior, and which are not, taking into account the estimated reliability of the sensory environment (Feldman & Friston, 2010; Friston, 2010; Slagter & Moorselaar, 2021).

In conclusion, the present study identifies statistical volatility as a determining factor of the brain’s ability to learn about, combine, and exploit task-relevant features in multiple dimensions to efficiently guide attention during search. As contextual uncertainty also varies as a function of search set size, and the number of search items is often manipulated within experiments and typically varies across studies of visual search, it is important that researchers consider this in future designs (Wöstmann et al., 2022). Moreover, the present findings suggest that in search tasks in which the colours of the targets and non-singletons are the same and predictable, global feature enhancement may enhance task performance in the face of distraction, next to or rather than singleton suppression.

## Data Availability

The data from this study are available via the Open Science Framework at: 10.17605/OSF.IO/DE78J

## Code availability

The code from this study is available via the Open Science Framework at: 10.17605/OSF.IO/DE78J
